# The successful uptake and sustainability of rapid infectious disease and antimicrobial resistance point-of-care testing requires a complex ‘mix-and-match’ implementation package

**DOI:** 10.1007/s10096-019-03492-4

**Published:** 2019-02-02

**Authors:** John P. Hays, Konstantinos Mitsakakis, Saturnino Luz, Alex van Belkum, Karsten Becker, Ann van den Bruel, Stephan Harbarth, John H. Rex, Gunnar Skov Simonsen, Guido Werner, Valentina Di Gregori, Gerd Lüdke, Tjeerd van Staa, Jacob Moran-Gilad, Till T. Bachmann, on behalf of the JPIAMR AMR-RDT consortium

**Affiliations:** 1 Na-908, Department of Medical Microbiology & Infectious Diseases, Erasmus University Medical Centre (Erasmus MC), Postbus 2040, 3000 CA Rotterdam, Netherlands; 2 Hahn-Schickard, Georges-Koehler-Allee 103, 79110 Freiburg, Germany; 3 Usher Institute of Population Health Sciences & Informatics, The University of Edinburgh, 9 Little France Road, Edinburgh, EH16 4UX UK; 4 BioMérieux Data Analytics Unit, 3 Route de Port Michaud, 38390 La Balme Les Grottes, France; 5 University Hospital Münster, DGHM, Münster, Germany; 6 Department of Primary Care Health Sciences, University of Oxford, Oxford, UK; 7 Infection Control Program, WHO Collaborating Center for Patient Safety, Geneva University Hospitals and Faculty of Medicine, Geneva, Switzerland; 8 F2G Ltd., Eccles, Manchester, UK; 9 University of Tromsø, Tromsø, Norway; 10 Robert Koch Institute, Wernigerode Branch, Wernigerode, Germany; 11 EUPHA (European Public Health Association), Otterstraat 118-124, Postbox 1568, 3500 BN Utrecht, The Netherlands; 12 GVM Care and Research, (Presidio San Pier Damiano- Faenza-), Corso Garibaldi 11, 48022 Lugo, Ravenna Italy; 13 Curetis GmbH, Holzgerlingen, Germany; 14 University of Manchester, Manchester, UK; 15 Dept. of Health Policy and Management, School of Public Health, Faculty of Health Sciences, Ben-Gurion University of the Negev, Beer Sheva, Israel; 16 The ESCMID Study Group for Genomic and Molecular Diagnostics (ESGMD), Basel, Switzerland; 17 Division of Infection and Pathway Medicine, Edinburgh Medical School: Biomedical Sciences, University of Edinburgh, Chancellor’s Building, 49 Little France Crescent, Edinburgh, EH16 4SB UK

**Keywords:** Point-of-care testing, Infectious diseases antimicrobial resistance, Implementation package, Diagnostic innovator, Healthcare providers, General public, Future proofing

## Abstract

The emergence and spread of antimicrobial resistance is one of the major global issues currently threatening the health and wealth of nations, with effective guidelines and intervention strategies urgently required. Such guidelines and interventions should ideally be targeted at individuals, communities, and nations, requiring international coordination for maximum effect. In this respect, the European Joint Programming Initiative on Antimicrobial Resistance Transnational Working Group ‘Antimicrobial Resistance - Rapid Diagnostic Tests’ (JPIAMR AMR-RDT) is proposing to consider a ‘mix-and-match’ package for the implementation of point-of-care testing (PoCT), which is described in this publication. The working group was established with the remit of identifying barriers and solutions to the development and implementation of rapid infectious disease PoCT for combatting the global spread of antimicrobial resistance. It constitutes a multi-sectoral collaboration between medical, technological, and industrial opinion leaders involved in in vitro diagnostics development, medical microbiology, and clinical infectious diseases. The mix-and-match implementation package is designed to encourage the implementation of rapid infectious disease and antimicrobial resistance PoCT in transnational medical environments for use in the fight against increasing antimicrobial resistance.

## Introduction

Infectious diseases are one of the major contributors to global morbidity and mortality. Further, the worldwide spread of antimicrobial resistance means that this burden is steadily increasing on a global scale, with the possibility of untreatable infectious diseases being commonplace in the near future [1]. Therefore, internationally coordinated efforts are required to find, describe, and implement effective global healthcare strategies that will help combat the threat of untreatable infectious diseases. In this respect, the use of rapid infectious disease and antimicrobial resistance point-of-care testing (PoCT) can be a key tool in tackling the global burden of infectious disease and limit the emergence and global spread of antimicrobial resistant microorganisms. Further, in this publication, the authors suggest that a ‘mix-and-match’ implementation package is required in order to ensure the most effective and efficient uptake and sustainability of rapid infectious disease and antimicrobial resistance PoCT. The term ‘implementation’ as used in this publication relates to a number of issues, including positively influencing clinical decision-making processes, helping inform patients on how to change their current behaviour, and taking note of healthcare economics, whereby healthcare providers expect ‘value for money’ with respect to the detection and treatment of infectious disease. A PoCT ‘mix-and-match implementation package’ is defined as an implementation package that offers a mixture of recommendations that can be individually chosen to best match the needs of healthcare providers, technology innovators, and the general public, whilst helping to ensure the sustainability (future-proofing) of rapid infectious disease and antimicrobial resistance PoCT.

## Mix-and-match implementation package for healthcare providers

Healthcare providers (including institutions such as hospitals, clinics, and emergency rooms, as well as individual clinical professionals such as doctors and nurses) are the primary potential users of rapid infectious disease and antimicrobial resistance PoCT, having direct access to patients and making empirical decisions about the actual need for a particular diagnostic test.

Healthcare providers should be encouraged to establish their own PoCT ‘working groups’, with the responsibility for establishing a policy for the implementation of rapid infectious disease and antimicrobial resistance PoCT into their own particular healthcare setting [2]. These working groups would also provide a focal point for the generation and distribution of point-of-care educational material to medical personnel. This will help to generate and maintain quality standards, for example, compliance with ISO 22870 accreditation. The working group should consider making a five point pre-implementation plan: (1) surveying information on perceived and real advantages and disadvantages of point-of-care testing within their own institution; (2) proactively developing contacts with point-of-care device innovators, national reimbursement groups, health technology assessment agencies, etc.; (3) establishing selection criteria for PoCT devices; (4) establishing institutional-wide management, quality control, and quality assurance procedures; and (5) promoting a culture of cost-benefit and cost-effective analysis, thereby justifying the actual implementation of PoCT, including rapid infectious disease and antimicrobial resistance PoCT. The working group should ideally contain experts from the institution’s own diagnostic laboratories as well as representatives of medical end-users, information technologists, pharmacists, finance, and patients. Such PoCT working groups could also act as a focal contact point for the introduction of expert knowledge into community healthcare settings.

On the level of antibiotic use, antibiotic stewardship teams (A-Teams) have been installed in American and European hospitals and conventionally include experts in internal medicine, microbiology, pharmacy, quality management, paediatrics, and intensive care (as needed). These teams work closely with infection prevention specialists in reducing the inappropriate use of antibiotics within medical institutions. However, the added value of rapid infectious disease and antimicrobial resistance PoCT to the antibiotic stewardship teams’ decision-making processes may not be fully appreciated, even though PoCT may provide an efficient approach for reducing the evolution and spread of antimicrobial resistance [3]. Here, further education of the respective stakeholders is required, as well as the addition of qualified molecular diagnosticians to the antibiotic stewardship teams. Once implemented, it would be ideal if the A team were easily available, e.g. by telephone, to answer questions from clinicians and nurses regarding AMR and AMR diagnostics.

At the individual level, the necessary implementation changes required to make informed decisions on the use of rapid infectious disease and antimicrobial resistance PoCT can be simply focused on increasing the current knowledge of medical professionals about the subject via a wide variety of sources, including scientific publications, which in general will explain the lessons learnt from previously published interventional studies. For example, Chandler et al. studied the behavioural issues affecting the implementation of rapid malaria testing and treatment in northern Tanzania [4]. The study found that negative test results could lead to conflict situations if a negative result meant that the health worker did not fulfil the patient’s expectation of receiving malaria treatment. Further, such malaria test negative patients may be prescribed antibiotics leading to an increase in the untargeted use of antibiotics [5]. Similar issues were identified in a study of C-reactive protein (CRP) testing for lower respiratory tract infections in Europe. These issues could have been addressed by implementing online behaviour change interventions with instructions, training, and patient resources [6].

In a recent publication, Klein et al. employed psychology to investigate how the perceptions of clinicians influenced their clinical decision-making process within emergency departments [7]. Differences in the decision to prescribe antibiotics by emergency department clinicians could be related to two possibly conflicting perceptions. These were ‘Why not take a risk?’ and ‘Antibiotics may be harmful’. The results reported in this study suggested that interventions to reduce inappropriate prescribing should emphasise the possibility of serious side effects when prescribing antibiotics. Rapid infectious disease and antimicrobial resistance PoCT educational campaigns could therefore emphasise that prescribing antibiotics also carries a risk to the patient. However, whether such perceptions are common to physicians within different countries, cultures or educational levels remains to be determined.

Whatever the method used, perhaps the best approach to successfully implementing such testing is to avoid the learning or adoption of ‘bad behaviour’ in the first place. This process is for example addressed by *AMR DxC - The Antimicrobial Resistance Challenge* competition, an interdisciplinary initiative whereby multi-disciplinary teams of young medical students and early career scientists from different geographic regions come together to discuss and work collaboratively on medical diagnostic solutions. Cross-cutting interdisciplinary discussions are key aspects of such programs, whereby younger generations of clinicians, diagnostic innovators, social scientists, and device designers discuss and understand the current challenges associated with the development and implementation of rapid infectious disease PoCT to tackle antimicrobial resistance [8].

## Mix-and-match implementation package for rapid infectious disease and antimicrobial resistance PoCT innovators

PoCT innovators can play a major role in ensuring the successful adoption of infectious disease testing. As an example, there is a current emphasis on adapting and developing cutting edge technologies for infectious disease diagnostics without focusing on the actual clinical need for these technologies in the detection of infectious diseases and antimicrobial resistance. Therefore, innovators should establish bi-directional communication channels with end-users and other stakeholders. These groups should interact synergistically, forming a positive feedback loop, whereby the changing needs of the end-user generates adaptation in diagnostic device development. One method could include the use of online or email-based questionnaires containing targeted questions that are designed to shape the desired current and future performance characteristics and settings of the device, thereby contributing to the creation of specific target product profiles (TPP). However, ideally, a range of methods is required to comprehensively understand user participation, possibly including interviews, observational studies, and focus group meetings [9] For sustainability, rapid infectious disease and antimicrobial resistance PoCT innovators should obtain up-to-date information on infectious disease research and epidemiology via regular visits to scientific conferences, accessing horizon scanning reviews [10] or by asking for relevant information from insurance companies, professional societies, patient associations, health technology assessment bodies, regulatory bodies and global infectious disease alert systems such as Promed (https://www.promedmail.org/).

Another large problem faced by innovators is the need for strong evidence to confirm that their technologies can actually improve current clinical practice, using *evidence-based* rather than *empirical* medicine. In this context, *evidence-based* relates to “the conscientious, explicit, and judicious use of current best evidence in making decisions about the care of individual patients”, and *empirical* to “based on, concerned with, or verifiable by observation or experience rather than theory or pure logic” [11] (https://en.oxforddictionaries.com/definition/empirical). The successful implementation of rapid infectious disease and antimicrobial resistance PoCT will rely on clinicians obtaining data from patient-centred studies that compare and contrast the new diagnostic test with current gold standard testing methods, considering inputs such as the costs of training, devices, test kits, quality control, and assurance schemes, as well as outputs such as reduced antibiotic prescribing costs, shorter hospitalisation, reduced antimicrobial resistance, and improved quality of life [12]. To generate these data, clinical studies need to be performed and the results published in scientific journals. Further, summaries or short information leaflets could form part of sales and marketing campaigns to build user awareness. That said, it is appreciated by the JPIAMR AMR-RDT working group that establishing and conducting these studies can be very expensive, especially for innovators in small and medium-sized enterprises. However, being trustworthy and open with consumers, healthcare authorities, investors, and patients would help avoid prominent crises, such as occurred with Theranos [13].

For further guidance, PoCT innovators should consult regulatory frameworks such as the latest In Vitro Diagnostic Device Regulation (EU 2017/746) of the European Union which also lays the legal framework for the European database on medical devices (EUDAMED) which by 2020 will facilitate access to information on existing diagnostics (http://eur-lex.europa.eu/legal-content/EN/TXT/?uri=CELEX%3A32017R0746). To succeed with the development of viable products, innovators need to consider business-to-business implementation strategies. Ultimately, innovators need to be able to invest in manufacturing facilities, salaries, marketing, etc., and clinicians need to be able to use PoCT with confidence of an improved health economic benefit.

A lack of standardised national and international reimbursement schemes is currently reducing the potential implementation and impact of PoCT. This effect is not only restricted to traditional healthcare settings, but also involves potential new markets such as home-based care, telemedicine, and in-home testing and monitoring, where some form of reimbursement is essential [14]. In some countries, clinicians are funded via a national healthcare service, which may be prepared to pay for in-house medical testing, for example for PoCT performed at a general practitioner’s office [15]. However, though patient healthcare insurance may be available in some countries, not everyone can afford to pay basic healthcare insurance premiums, and a choice may have to be made between paying for a PoCT or simply buying antibiotics. In this respect, evidence of added value is essential and innovators could encourage the implementation of their diagnostics by being flexible in their pricing strategy, for example, by basing their prices on the number of tests sold. Also, cooperation with product development partnerships and/or non-governmental organisations are other possible options for promoting sales and reimbursement, including for example the establishment of ‘Diagnostic Market Stimulus Pots’ to ‘ensure a market based revenue stream for developers’ [16]. In any case, finding funding for healthcare validation studies is one of the major challenges facing all developers of healthcare diagnostics.

Gender and cultural issues may also play a major role in preventing the implementation of PoCT due to the stigmatisation of patients found to be infected with a particular pathogen—not forgetting that stigmatisation could also be based on a false positive test result. For example, a positive human immunodeficiency virus, sexually transmitted disease, Ebola, etc., test result may be utilised to justify existing prejudices or beliefs based on gender, sexual preference, or immigration status [17]. Further, Borg identified behaviour characteristics related to “cultures that are low in uncertainty avoidance and power distance, and high in individualism and masculinity” that could influence PoCT implementation strategies [18]. Whilst it is not feasible for, or indeed the responsibility of, diagnostics innovators to change an individual’s or culture’s beliefs or prejudices, innovators must be sympathetic and knowledgeable about their target population. In this respect, one possible route for accessing potentially stigmatised communities would be to approach the community via a trusted intermediary, who may be a community opinion leader, local tribal elder, or a religious leader.

If we consider the future expansion of healthcare, then it is not unreasonable to assume that some form of PoCT will eventually be routinely used by members of the public, via pharmacies or at home, without the supervision of a medical professional. To prevent confusion by the pharmacist or end-user, as well as unnecessary visits to already overworked family doctors, pharmacists, and emergency departments, PoCT innovators should ideally establish telephone or internet hotlines that are available to answer the questions of pharmacists or PoCT device home-based users. These hotlines could potentially form additional arms of existing medical information hotlines, for example the UK NHS 111 service (www.nhs.uk/NHSEngland/AboutNHSservices/Emergencyandurgentcareservices/Pages/NHS-111.aspx). Further, by collecting data from such services, hotline providers could generate essential feedback to diagnostics innovators that could be used to increase device performance or improve the simplicity of instruction material supplied with the diagnostic. However, it should be noted that performing PoCT at the pharmacy or at home is a controversial issue. For example, a recent pharmacy ‘test and treat’ scheme for sore throat diagnosis in England attracted some criticism regarding poor sensitivity and lack of a full cost-effectiveness analysis [19]. Finally, diagnostics innovators should gain a deep understanding of the context and views of potential end-users before product development progresses into a functional and marketable device, for example, using established ‘user-centred’, ‘participatory design’, or ‘person-based’ approaches. Innovators should not simply focus on the development of ‘technology for technology’s sake’. Just because a technology exists that could be adapted for PoCT, does not mean to say that the technology will ultimately be successful as a healthcare diagnostic.

## Mix-and-match implementation package for the general public

Promoting change in the standard practice of clinical medicine has traditionally been achieved by actively targeting clinicians, with the possible exception of vaccination campaigns that are designed to encourage the general public to vaccinate themselves and their children against a range of infectious diseases. However, the realisation that the world is approaching an antimicrobial resistance catastrophe where clinically relevant microorganisms are resistant to all available antibiotics has led to global efforts to educate the general public about the dangers of the misuse of antibiotics. These educational efforts need to include information on the value of PoCT in helping reduce untargeted antibiotic prescribing practices. In this respect, PoCT educational material/campaigns could be generated that are aimed at the general public via hospital and family doctor leaflets, radio/newspaper/TV advertisements, etc. Slogans adapted from existing infectious disease diagnostic companies or new slogans such as ‘Lack of a diagnostic result? Unnecessary antibiotics during the consult!’ could be used. Additionally, the general public should be encouraged to think about the consequences of antibiotic prescribing on themselves (for example the ‘collateral damage’ caused by antibiotics to the patient’s own protective microbiota) and on their own extended families and communities (for example the threat of growing worldwide antimicrobial resistance on communities in which their family members live). Such educational material should take into account the education level, geography, and religious belief of the target audience.

Perhaps health warnings could be added to packets of antibiotics that would indicate that antibiotics should ideally only be taken after a positive (PoCT) test result has been obtained [20]. Such warnings may help generate a mental link between a lack of accurate diagnosis and the incorrect/over-prescribing of antibiotics. Such a link could persuade patients to discuss diagnostic issues with their clinician before receiving antibiotics. However, it should be noted that in order to be effective, the content of such health warnings should address the patients’ own beliefs, for example, a belief that they themselves are not at risk from antimicrobial resistant infections or that their behaviour may not be contributing to the increase and spread of antimicrobial resistance.

## Mix-and-match implementation considerations for long-term viability (future proofing)

The long-term viability of PoCT may be affected by, for example: (1) genomic mutations in pathogenic microorganisms; (2) the emergence of new antimicrobial resistance traits; (3) antigenic shifts related to the introduction of new vaccines; (4) the spread of previously unknown pathogenic microorganisms to humans and domesticated animals, and (5) the creation of new ecological niches due to global climate change. Therefore, regular adaptation of diagnostic devices (future proofing) will be necessary to ensure that their added value is actually sustainable. Accurately predicting the future is always difficult, but armed with a few simple concepts (described below), both innovators and end-users may be able to adapt their current behaviour so as to plan for, and reap, the potential rewards available from the long-term viability of PoCT.

PoCT innovators and (inter)national health authorities should be aware that decentralisation may bring with it new (exploitable) possibilities. For example, the use of telemedicine may facilitate the interpretation of results by clinicians far away from the site where a patient is being tested, as well as the ability to collect global infectious disease test results in real time during disease outbreaks. However, these exploitable possibilities bring with them potential problems such as patient authorisation, investment in large-scale data storage centres and management, data security, and ethical issues.

Smartphone ownership is ubiquitous and the rate of smartphone ownership in emerging economies and developing countries has been increasing at an extraordinary rate. This rise in the ownership of smartphones has been accompanied by a rise in the development of both software and hardware applications in the field of rapid PoCT diagnostics, for example, the Colorimetrix app (portable spectrophotometer), the smartphone ‘Olloclip’ lens (microscopy), and smartphone ‘Otoscope’ (ear infections). Additionally, large steps are being made in the rapid nucleotide sequencing of pathogens using portable devices, facilitating the generation of so-called ‘omics’-based data including genomics and transcriptomics data (https://nanoporetech.com/). Further, although possible discrepancies between genomic and phenotypic data currently exist, future advances in the transcriptomic sequencing of mRNA transcripts may provide the necessary link between genomic data and phenotypic characteristics of infectious disease pathogens [21].

As ‘omics-based’ data becomes more abundant, it is expected that machine learning (i.e. the ability of computers to learn without being explicitly programmed to do a task), will become increasingly relevant to behaviour change in clinical settings. In this respect, clinical decision support systems (CDSS) incorporated in, or connected to, PoCT diagnostics may help clinicians to (1) distinguish between viral and bacterial infections; (2) assess regional and personalised antimicrobial resistance profiles; (3) improve antibiotic prescribing practices, and (4) predict a patient’s response to treatment [22]. However, the integration of machine learning into clinical practice requires a combination of accuracy, predictive power, and interpretability. This will require the establishment of interdisciplinary expert panels and clinical trials, rather than simply relying on computer technologists to write algorithms and publish their results. That said, the importance of artificial intelligence in the detection and validation of clinical biomarkers in infectious disease diagnosis has been highlighted by the winner of the XPRIZE (https://tricorder.xprize.org/prizes/tricorder/articles/family-led-team-takes-top-prize-in-qualcomm-tricor). The winner recently received $2·6 million for an artificial intelligence-based engine ‘DxtER’, which consists of “a group of non-invasive sensors that are designed to collect data about vital signs, body chemistry and biological functions. This information is then synthesised in the device’s diagnostic engine to make a quick and accurate assessment”. This type of development points to a future where individual PoCT diagnostics may become redundant to new more powerful diagnostics that are able to diagnose many different infectious and non-communicable diseases together and potentially predict treatment outcomes in a more cost-effective manner.

Decentralisation and the ability to transmit data over vast distances, whilst simultaneously being able to link many thousands of diagnostic devices with each other, could be profoundly advantageous for the health of citizens and nations. The worldwide implementation and connectivity of PoCT diagnostics could lead to advances in holistic healthcare monitoring, with such devices regularly communicating with each other—exchanging patient information, clinical histories, cardiology data, X-rays, tomography results, etc. Additionally, by blending the results of such data with data collected from environmental, ecological, meteorological, and entomological sources, new multi-dimensional algorithms could be developed for high-level predictive modelling of the origin and spread of existing and emerging infectious diseases. Such global healthcare networks and algorithms could also act as early warning systems for the rapid detection of epidemics, allowing the targeted distribution of healthcare resources to affected areas [23]. Examples include (1) modelling and predicting the effect of global warming and urbanisation on infectious diseases, such as the spread of extensively drug-resistant tuberculosis in HIV populations; (2) monitoring the spread of mosquito borne diseases from Africa into Europe; (3) determining the genotype of currently circulating strains of influenza in their seasonal progression around the world; and (4) monitoring the health of migrant and refugee populations. To be feasible, standardised data collection, databases, data exchange, privacy protocols, and cybersecurity issues will have to be implemented, especially in an age of connectivity via the *Internet of Things*. However, it should be noted that saving costs on data security in an age of PoCT diagnostic connectivity could potentially result in poorly protected devices with vulnerabilities that leave them open to hacking and malware (www.theregister.co.uk/2015/10/19/bods_brew_ikettle_20_hack_plot_vulnerable_london_pots/).

**Fig. 1:**
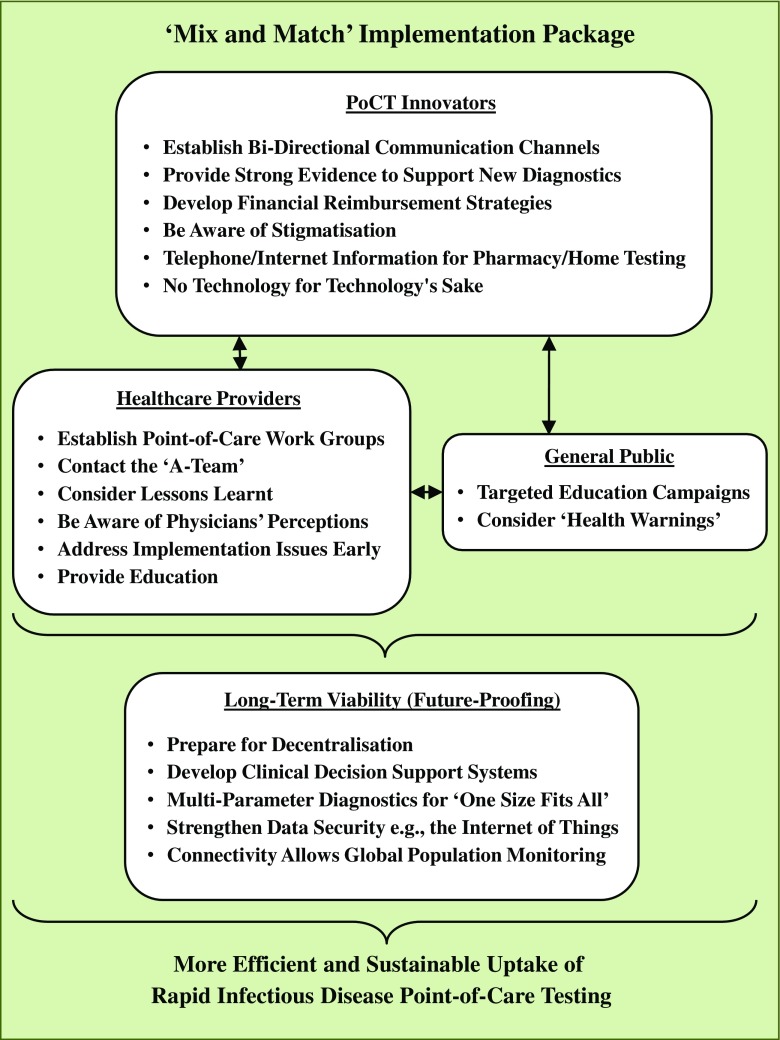
Complex ‘mix-and-match’ implementation package for the successful implementation of rapid infectious disease and antimicrobial resistance point-of-care testing. One barrier to the successful uptake and sustainability of rapid infectious disease and antimicrobial resistance point-of-care testing (PoCT) is the need to take into account the long-term viability, sustainability, and durability of these diagnostics. In this respect, the implementation components in the figure may be chosen using a ‘mix-and-match’ process to best suit individual healthcare settings and/or individual rapid infectious disease and antimicrobial resistance PoCT diagnostic operating characteristics. ‘A-Team’ = Antibiotic Stewardship Team. ‘Internet of Things’ = the network of devices, e.g. home appliances that contain electronics and software which allows these devices to connect, interact, and exchange data

The complex ‘mix and match’ implementation package is shown in Fig. 1.

## Conclusions

Infectious diseases are one of the major contributors to global morbidity and mortality. In this respect, the successful implementation of rapid PoCT into healthcare settings has the potential to help slow down and prevent the global spread of infectious diseases and antimicrobial resistances. This can be achieved by better monitoring of infections and by facilitating the accurate and targeted prescribing of antibiotics to those patients who actually need them. However, it should be noted that the successful implementation and long-term viability of rapid infectious disease and antibiotic resistance PoCT is not solely dependent on the development, sales, and marketing of diagnostic devices. Instead, a more sophisticated and holistic approach is required that takes the opinions and requirements of end-users into account, whilst remaining technologically flexible in order to meet the demands of future trends. Consideration of the ‘mix-and-match’ implementation change package described in this publication will help facilitate the uptake and sustainability of PoCT diagnostics into healthcare settings. Successful implementation will be a key step in reducing the spread and development of antibiotic resistant infections, helping improve global healthcare outcomes in terms of patient morbidity and mortality.
